# Chronic kidney disease associated with decreased bone mineral density, uric acid and metabolic syndrome

**DOI:** 10.1371/journal.pone.0190985

**Published:** 2018-01-10

**Authors:** Bo-Lin Pan, Song-Seng Loke

**Affiliations:** Department of Family Medicine, Kaohsiung Chang Gung Memorial Hospital, Niaosong District, Kaohsiung, Taiwan; The Pennsylvania State University, UNITED STATES

## Abstract

**Objective:**

The relationship between decreased bone mineral density (BMD) and chronic kidney disease (CKD) is controversial. The associations among metabolic syndrome (MetS), serum uric acid and CKD are also unclear. We aimed to investigate the relationship between decreased BMD, MetS, serum uric acid and CKD in a general population.

**Methods:**

A total of 802 subjects who visited a medical center in Southern Taiwan and underwent a BMD measured by dual-energy X-ray absorptiometry (DEXA) during a health examination were enrolled in this retrospective cross-sectional study. Either osteopenia or osteoporosis was defined as decreased BMD. CKD was defined as the estimated glomerular filtration rate (eGFR) of less than 60 mL/min/1.73m^2^. Simple and multivariate logistic regression analyses were used to investigate the association between variables, decreased BMD and CKD.

**Results:**

Of the 802 subjects with a mean age of 54.4±10.2 years, the prevalence of decreased BMD was 62.9%, and CKD was 3.7%. Simple logistic analysis showed that sex (OR 3.50, 95% CI 1.21–10.12, p = 0.021), age (OR 1.14, 95% CI 1.07–1.21, p<0.001), BMI (OR 1.11, 95% CI 1.01–1.22, p = 0.028), waist circumference (OR 1.06, 95% CI 1.02–1.10, p = 0.002), SBP (OR 1.03, 95% CI 1.01–1.04, p = 0.003), DBP (OR 1.03, 95% CI 1.00–1.06, p = 0.030), HDL-C (OR 0.97, 95% CI 0.94–1.00, p = 0.026), uric acid (OR 1.84, 95% CI 1.49–2.27, p<0.001), metabolic syndrome (OR 2.68, 95% CI 1.29–5.67, p = 0.009), and decreased BMD (OR 3.998, 95% CI 1.38–11.57, p = 0.011) were significantly associated with CKD. Multivariate analysis showed that age (OR 1.05, 95% CI 1.03–1.07, p<0.001), decreased BMD (OR 0.64, 95% CI 0.45–0.91, p = 0.013), and uric acid (OR 1.40, 95% CI 1.24–1.59, p<0.001) were significantly independently associated with CKD.

**Conclusions:**

Decreased BMD, uric acid and MetS were significantly associated with CKD.. Further large and prospective cohort studies are necessary to investigate whether management of osteoporosis, hyperuricemia, or MetS might prevent the progression of CKD.

## Introduction

Chronic kidney disease (CKD) is a global health problem and increasing worldwide. The prevalence of CKD in Taiwan was 9.8–11.9% and 13.1% in the United States by National Health and Nutrition Examination Survey (NHANES III, 1999–2004) [1, 2]. The prevalence of CKD stage 3–5 or total CKD is approximately 12.9–15.1% in Japan, 3.2–11.3% in China, 7.2–13.7% in Korea, based on different published reports [1].

Osteoporosis is a silent health problem, which is characterized by decreased bone mineral density (BMD) with a risk of spine and hip fractures. Osteoporosis-related mortality and disability result in adverse impact on patients, their families, society and the health system. End-stage renal disease (ESRD) is significantly associated with decreased BMD and osteoporosis [3]; however, the relationship between decreased BMD and CKD is controversial. A study in cross-sectional analysis showed association between renal function and BMD, and this was strongest at higher CKD stages [4]. However from the Third National Health Assessment and Nutritional Examination Survey (NHANES III) data, there was no significant relationship of decreased BMD and CKD after adjustment for age, sex and race [5]. Recently, the Kidney Disease: Improving Global Outcomes (KDIGO) publication suggests BMD testing can play a role in screening among the population with stages 3–5 CKD [6]. Several studies have revealed the association of renal function and BMD in Asian countries [7, 8], but there is a lack of such studies performed in Taiwan.

Metabolic syndrome (MetS) is a complicated disorder including several cardiovascular risk factors composed of abdominal obesity, high blood pressure, hyperglycemia, hypertriglyceridemia and low high-density lipoprotein cholesterol (HDL-C). MetS has been related to several diseases, such as cardiovascular disease, diabetes mellitus, fatty liver disease, and even with increased all-cause mortality [9]. MetS is also associated with the renal injury in previous studies [10, 11]. In addition, there is a correlation between MetS and hyperuricemia in increasing the risk of cardiovascular disease and mortality [12, 13]. Hyperuricemia and MetS also cause risk for CKD through similar mechanisms such as endothelial dysfunction, oxidative stress and systemic inflammation [13, 14].

No previous study has assessed the association between BMD, serum uric acid, MetS, and CKD. Therefore, we aimed to investigate the association between BMD, serum uric acid, MetS, and CKD in a healthy population in Taiwan.

## Methods

This retrospective cross-sectional study was performed by using the data from the health examination database in a medical center of the southern Taiwan. All data were fully anonymized before access from the computer database and the ethics committee waived the requirement for informed consent. We included subjects who had been assessed for renal function, uric acid, metabolic factors and had undergone dual energy X-ray absorptiometry scan (DEXA) from January, 2012 to December, 2013. The study was approved by the Institutional Review Board and the Ethics Committee of Chang Gung Memorial hospital.

The medical records revealed sex, age, body weight, height, waist circumstance, lipid profile, fasting blood glucose, serum uric acid, blood pressure, and BMD. Body mass index (BMI) was calculated as the weight in kilograms divided by the height in meters squared.

### Determination of bone mineral density

Bone mineral density (BMD) was measured using a GE Lunar bone densitometer. The *T*-score is the number of standard deviations by which a given measurement differs from the mean for a normal young adult reference population. According to the World Health Organization definition [10], osteoporosis is defined as *T*-score ≤ −2.5, and the osteopenia is defined as a T-score between −1 and −2.5. Decreased BMD in the present study included osteoporosis and osteopenia.

### Blood biochemistry measurements and biometric parameters

Blood was collected by venipuncture after an overnight fast. Routine serum chemistry determinations were measured by standard automated techniques. The estimated glomerular filtration rate (eGFR) was used for assessment of renal function. The eGFR was calculated by the Modification of Diet in Renal Disease (MDRD) equation. A simplified MDRD equation was used to estimate GFR (mL/ min/1.73m^2^) = 175×[serum creatinine]^−1.154^ x [age]^−0.203^ x (0.742 if female) x (1.212 if African American). CKD in our study was defined as eGFR being less than 60 mL/ min/1.73m^2^, indicating stage 3 CKD according to the National Kidney Foundation staging system used in the Chinese population in Taiwan [11].

Fasting blood glucose, serum uric acid, serum total cholesterol, low-density lipoprotein cholesterol (LDL-C), and high-density lipoprotein cholesterol (HDL-C), and triglycerides (TG), were also measured by automatic biochemistry analyzer.

### Definition of metabolic syndrome

The criteria of MetS were set by a joint interim statement of the International Diabetes Federation Task Force on Epidemiology and Prevention; American Heart Association; Word Heart Federation; International Atherosclerosis Society; National Heart, Lung and Blood Institute; and International Association for the Study of Obesity [15]. The MetS was diagnosed as the presense of at least three of the following five components: (1) high blood pressure (≥130/85 mmHg or under treatment); (2) elevated triglycerides (≥150 mg/dL); (3) reduced HDL-C (< 40 mg/dL in men or <50 mg/dL in women); (4) high fasting serum glucose (≥ 100 mg/dL or under treatment); and (5) elevated waist circumference (≥90 cm in men and ≥80 cm in women).

### Statistical analysis

All data are described as the mean ± standard deviation for continuous variables and as numbers and percentages for categorical variables. SPSS software version 19.0 (IBM Corp., Armonk, NY, USA) was used for the statistical analysis. The characteristics of subjects were compared by *χ*^*2*^-test for the categorical variables and Student’s t-test for the continuous variables. Simple logistic regression analysis was used for the odds ratio (OR) for CKD. Variables that were significant in simple logistic regression analysis were entered into the multivariate logistic regression analysis. ORs were presented with 95% confidence intervals (CI). A P-value of <0.05 was considered statistically significant.

## Results

We initially enrolled 904 subjects who underwent BMD and biochemistry blood examinations. One hundred and two subjects were excluded from the final analysis due to missing data in some biochemical variables. Finally, 802 subjects were included in the statistical analysis. The mean age of all subjects was 54.4±10.2 years and of the female and male subjects mean ages were 55.2±9.7 and 54.0±10.4 years respectively.

Table 1 compares the baseline characteristics of the female and male subjects. The body height, body weight, BMI, waist circumference, diastolic blood pressure (DBP), fasting blood glucose, total cholesterol, HDL-C, TG, uric acid, the number of CKD and MetS between females and males had significant difference, but age, systolic blood pressure (SBP), BMD, and LDL-C between females and males did not demonstrate significant difference.

**Table 1:** Baseline characteristics.

	All(n = 802)	Female(n = 274)	Male(n = 528)	P value
Age(years)	54.4±10.2	55.2±9.7	54.0±10.4	0.098
Body Height(cm)	165.2±8.3	156.9±5.3	169.5±6.1	<0.001*
Body Weight(Kg)	68.3±12.7	58.1±9.4	73.6±10.7	<0.001*
BMI(Kg/m^2^)	24.9±3.6	23.6±3.8	25.6±3.3	<0.001*
Waist circumference(cm)	84.9±10.2	78.2±9.4	88.4±8.8	<0.001*
SBP(mmHg)	130.6±19.3	129.0±21.8	131.4±17.8	0.094
DBP(mmHg)	83.7±14.9	80.2±15.7	85.5±14.1	<0.001*
BMD				0.096
Normal, n, %	298(37.1)	91(33.2)	207(39.2)	
Decreased, n, %	504(62.9)	183(66.8)	321(60.8)	
Blood glucose(mg/dl)	99.5±24.3	96.9±25.4	100.9±23.6	0.026*
HDL cholesterol(mg/dl)	56.3±15.5	65.6±16.1	51.4±12.8	<0.001*
Triglyceride(mg/dl)	133.4±85.9	107.5±61.1	146.9±93.6	<0.001*
Total cholesterol(mg/dl)	195.9±85.9	202.5±35.6	192.6±35.4	<0.001*
LDL cholesterol(mg/dl)	113.7±32.4	115.6±32.0	112.7±32.6	0.234
Uric acid(mg/dl)	6.2±1.5	5.33±1.28	6.71±1.41	<0.001*
CKD(eGFR<60), n, %	30(3.7)	4(1.5)	26(5.0)	0.014*
MetS, n, %	247(30.8)	63(23.0)	184(34.8)	0.001*

*: Indicates a significant difference, *p* < 0.05.

Abbreviations: BMI, body mass index; LDL, low-density lipoprotein; HDL, high-density lipoprotein; CKD, chronic kidney disease; BMD, bone mineral density; eGFR, estimated glomerular filtration rate; SBP, systolic blood pressure; DBP, diastolic blood pressure; MetS, metabolic syndrome.

The differences between subjects with CKD and non-CKD are presented in Table 2. When compared with non-CKD, subjects with CKD were significantly older, a higher percentage were males, had decreased BMD and MetS, higher BMI, waist circumference, DBP and uric acid measures.

**Table 2:** Comparison between subjects with CKD and non-CKD.

	CKD(n = 30)	Non-CKD(n = 772)	P value
Sex			0.014*
Male, n, %	4(13.3)	502(65.0)	
Female, n, %	26(86.7)	270(35.0)	
Age(year)	62.5±6.7	54.1±10.1	<0.001*
Body Height(cm)	164.4±5.2	165.2±8.4	0.410
Body Weight(Kg)	71.1±8.4	68.2±12.8	0.072
BMI(Kg/m^2^)	26.3±2.8	24.9±4.0	0.028*
Waist circumference(cm)	90.6±8.9	83.7±10.2	0.002*
SBP(mmHg)	140.8±17.5	130.2±19.5	0.083
DBP(mmHg)	89.1±8.4	83.5±15.0	0.041*
Blood glucose(mg/dl)	101±16.1	99.5±24.5	0.646
HDL cholesterol(mg/dl)	50.0±11.4	56.5±15.6	0.025*
Triglyceride(mg/dl)	146.0±67.9	132.9±86.5	0.413
Total cholesterol(mg/dl)	187.4±33.1	196.3±35.8	0.183
LDL cholesterol(mg/dl)	108.2±31.7	113.9±32.4	0.343
Uric acid (mg/dl)	8.0±1.33	6.2±1.48	<0.001*
Decreased BMD, n, %	26(86.7)	478(61.9)	0.006*
MetS, n, %	16(53.3)	231(30.0)	0.006*

*: Indicates a significant difference, *p* < 0.05

Abbreviations: BMI, body mass index; LDL, low-density lipoprotein; HDL, high-density lipoprotein; CKD, chronic kidney disease; BMD, bone mineral density; eGFR, SBP, systolic blood pressure; DBP, diastolic blood pressure; MetS, metabolic syndrome.

The results from the simple and multiple stepwise logistic regression analyses of variables associated with CKD are presented in Tables 3 and 4 respectively.

**Table 3:** Simple logistic regression analysis for association of CKD with different variables.

	OR (95% CI)	p value
Sex	3.50(1.21–10.12)	0.021*
Age	1.14(1.07–1.21)	<0.001*
BMI	1.11(1.01–1.22)	0.028*
Decreased BMD	3.998(1.38–11.57)	0.011*
Waist circumference	1.06(1.02–1.10)	0.002*
SBP	1.03(1.01–1.04)	0.003*
DBP	1.03(1.00–1.06)	0.030*
Blood glucose	1.00(0.99–1.02)	0.646
HDL cholesterol	0.97(0.94–1.00)	0.026*
Triglyceride	1.00(0.998–1.01)	0.413
Total cholesterol	0.99(0.98–1.00)	0.182
LDL cholesterol	0.99(0.98–1.01)	0.342
Uric acid	1.84(1.49–2.27)	<0.001*
MetS	2.68(1.29–5.67)	0.009*

*: Indicates a significant difference, *p* < 0.05

Abbreviations: BMI, body mass index; LDL, low-density lipoprotein; HDL, high-density lipoprotein; CKD, chronic kidney disease; BMD, bone mineral density; SBP, systolic blood pressure, DBP, diastolic blood pressure; MetS, metabolic syndrome.

**Table 4:** Multiple stepwise logistic regression analysis for association of CKD with different variables.

	OR(95% CI)	p value
Sex	1.48(0.97–2.26)	0.71
Age	1.05(1.03–1.07)	<0.001*
BMI	0.98(0.89–1.07)	0.97
Decreased BMD	0.64(0.45–0.91)	0.013*
Waist circumference	0.98(0.95–1.02)	0.304
SBP	1.01(0.99–1.04)	0.417
DBP	1.01(0.97–1.04)	0.776
HDL cholesterol	1.00(0.98–1.01)	0.391
Uric acid	1.40(1.24–1.59)	<0.001*
MetS	1.18(0.77–1.79)	0.444

*: Indicates a significant difference, *p* < 0.05

Abbreviations: BMI, body mass index; HDL, high-density lipoprotein; CKD, chronic kidney disease; BMD, bone mineral density; SBP, systolic blood pressure, DBP, diastolic blood pressure; MetS, metabolic syndrome.

In simple logistic regression analysis, sex (OR 3.50, 95% CI 1.21–10.12, p = 0.021), age (OR 1.14, 95% CI 1.07–1.21, p<0.001), BMI (OR 1.11, 95% CI 1.01–1.22, p = 0.028), waist circumference (OR 1.06, 95% CI 1.02–1.10, p = 0.002), SBP (OR 1.03, 95% CI 1.01–1.04, p = 0.003), DBP (OR 1.03, 95% CI 1.00–1.06, p = 0.030), HDL-C (OR 0.97, 95% CI 0.94–1.00, p = 0.026), uric acid (OR 1.84, 95% CI 1.49–2.27, p<0.001), MetS (OR 2.68, 95% CI 1.29–5.67, p = 0.009), and decreased BMD (OR 3.998, 95% CI 1.38–11.57, p = 0.011) were significantly associated with CKD. In multiple stepwise logistic regression analysis, age (OR 1.05, 95% CI 1.03–1.07, p<0.001), decreased BMD (OR 0.64, 95% CI 0.45–0.91, p = 0.013), and uric acid (OR 1.40, 95% CI 1.24–1.59, p<0.001) were significantly independently associated with CKD.

## Discussion

In the present retrospective cross-sectional study, the main finding was that decreased BMD, and hyperuricemia were independently significantly associated with CKD in a healthy Taiwanese population. MetS and its components, such as blood pressure and HDL-C were associated with CKD, but not independently.

The prevalence of decreased BMD was significantly higher in the CKD group (86.7%) than in the non-CKD group (61.9%). As for the relationships between renal function and BMD, Jassal *et al*. revealed that renal function was significantly associated with BMD. On the other hand, those with lower renal function are at higher risk for osteoporosis [4]. The other two small studies also reported the relationships between the creatinine clearance and BMD were significant for women [16,17]. In our study, the result also showed that decreased BMD was significantly associated with CKD.

CKD is related to many different mineral and bone disorders. Decreasing eGFR is associated with secondary hyperparathyroidism, abnormalities in 1,25-dihydroxy vitamin D synthesis, hyperphosphatemia, chronic metabolic acidosis, and elevated sclerotia and/or fibroblast growth factor 23 (FGF-23) levels, which results in the bone absorption and formation [18–21]. Another possible mechanism is that chronic metabolic acidosis due to decreased serum bicarbonate may result in bone resorption [22]. A study revealed an early impairment of trabecular microarchitecture in stage II-IV CKD patients. The study also showed early impairment of bony structures among early-stage CKD patients [23].

Decreased BMD in populations with CKD increases the risk of fracture. In 2017, KDIGO guidelines suggest BMD testing to assess fracture risk if results will impact treatment decisions [6]. However, the above CKD-related bone and mineral disorders might reduce the bone strength. Further alternative techniques to measure the bone strength, such as quantitative computed tomography (CT) or trabecular bone score based on DEXA might improve fracture prediction in CKD [24, 25].

Environmental factors and lifestyle may affect ethnic differences in bone mass. Because ethnic differences in bone density and CKD have been described [24], we can assume that the association between BMD and CKD might be different among ethnic groups. One cross-sectional study for a general Korean population revealed osteoporosis may be highly prevalent in individuals with moderate to severe CKD [7]. However, cross-sectional study of a Chinese population who received health examination showed mild-to-moderate CKD was not independently associated with decreased BMD after adjusting for confounding factors [25]. In this study, we found that decreased BMD was independently associated with CKD.

The relationship of hyperuricemia and CKD is inconsistent. There are many studies supporting hyperuricemia as a risk factor for CKD. A study of 167 Japanese patients with CKD (eGFR < 60 mL/min/1.73 m2) who received renal biopsy found that hyperuricemia was associated with renal arteriolar hyalinosis and wall thickening consistent with renal arteriolar damage after adjusting for age, sex, hypertension, diabetes, and eGFR [26]. The renal injury was caused by hyperuricemia which is involved in multiple mechanisms such as tubulointerstitial inflammation, endothelial cell dysfunction, fibrosis and renal arteriolopathy [27–29]. However, a cohort study showed no significant association between hyperuricemia and progression of kidney disease and development of kidney failure [30]. Our study showed that uric acid was independently associated with CKD. Further studies could emphasize whether the serum uric acid or other confounding factors are associated with the progression of kidney disease in larger populations.

MetS is a complicated disorder with the increasing risk of cardiovascular disease and all-cause mortality [9]. The main features of MetS include central obesity, elevated blood pressure, hyperglycemia, hypertriglyceridemia and low HDL-C. Some studies have suggested an association between MetS and CKD. One study with 6217 people of the general US population showed a significant association between MetS and CKD, suggesting that MetS may play a role in the cause of CKD [31]. Another study in Japan showed MetS was an independent risk factor of CKD in Japanese [32]. Yet a further study on the same topic conducted in Chinese enrolling 15160 Chinese adults revealed a significant relationship between the MetS and risk of CKD [33]. However, in our present study, MetS was correlated with CKD, but not independently because of other strong confounding factors.

Few studies have investigated the interaction between serum uric acid and MetS contributing to CKD [34,35]. In the present study, our findings demonstrated serum uric acid was independently associated with CKD despite MetS and its components. The similar pathophysiological mechanism causing glomerular and tubular fibrosis, such as endothelial dysfunction, oxidative stress, and systemic inflammation with cytokine synthesis might result from coexistence of hyperuricemia and MetS [14]. However, it is not clear whether treating MetS or hyperuricemia will prevent the progression of CKD and other complications. Therefore, further studies should emphasize that management of hyperuricemia and MetS might prevent renal impairment.

Our study has some limitations. First, the study subjects came from a single hospital, which might not be representative of other settings. Second, retrospective studies based on abstraction of medical records are constrained by the accuracy and completeness of the records. Third, we conducted this study with relatively healthy people from the database in the health examination, where the prevalence of CKD in the present population was only 3.7% which is much lower than the Taiwan national prevalence. Fourth, some laboratory data, such as intact parathyroid hormone (iPTH), serum calcium, serum phosphorus, serum specific alkaline phosphatase or serum vitamin D level were unavailable, because this study was retrospective from the health examination.

## Conclusions

Results from this retrospective study revealed a strong relationship between decreased BMD, uric acid and MetS with CKD in a general population in Taiwan. Further prospective cohort studies are necessary to investigate whether treating osteoporosis, hyperuricemia, or MetS might prevent the progression of CKD.
